# Mortality and major morbidities in very preterm infants born from assisted conception or naturally conceived: results of the area-based ACTION study

**DOI:** 10.1186/1471-2393-14-307

**Published:** 2014-09-05

**Authors:** Carlo Corchia, Monica Da Frè, Domenico Di Lallo, Luigi Gagliardi, Franco Macagno, Virgilio Carnielli, Silvana Miniaci, Marina Cuttini

**Affiliations:** International Centre on Birth Defects and Prematurity, ICBD, via Carlo Mirabello 14, 00195 Rome, Italy; Unit of Epidemiology, Regional Health Agency of Tuscany, Florence, Italy; Unit of Epidemiology, Regional Health Agency of Lazio, Rome, Italy; Woman and Child Health Department, Pediatrics and Neonatology Division, Versilia Hospital, Viareggio, Italy; Neonatal Intensive Care Unit, S. Maria della Misericordia University Hospital, Udine, Italy; Maternal and Child Health Institute, Marche University and Salesi Hospital, Ancona, Italy; Neonatal Intensive Care Unit, Pugliese-Ciaccio Hospital, Catanzaro, Italy; Unit of Epidemiology, Bambino Gesù Children’s Hospital, Rome, Italy

**Keywords:** Assisted conception, Preterm birth, Neonatal mortality, Neonatal morbidity, Singletons, Multiples

## Abstract

**Background:**

The use of assisted conception (AC) has been associated with higher risk of adverse perinatal outcome. Few data are available on the outcome of AC-neonates when pregnancy ends before 32 weeks of gestational age.

The aim of this study was to compare the short-term outcome of AC- and naturally conceived preterm infants <32 weeks gestation.

**Methods:**

The area-based cohort study ACTION collected data on births 22-31 weeks gestation occurred in 2003-05 in 6 Italian regions. Infants born to 2529 mothers with known mode of conception were studied. The main outcomes were hospital mortality and survival free from major morbidities (IVH grade 3-4, cPVL, ROP stage ≥3, BPD), and were assessed separately for single and multiple infants. Other outcomes were also investigated. Multivariable logistic analyses were used to adjust for maternal and infants’ characteristics. To account for the correlation of observations within intensive care units, robust variance and standard error estimates of regression parameters were computed.

**Results:**

AC was used in 6.4% of mothers. Infants were 2934; 314 (10.7%) were born after AC. Multiples were 86.0% among AC and 21.7% among non-AC babies. In multivariable analysis no statistically significant difference in hospital mortality and survival without major morbidities was found between AC and non-AC infants. The risk of BPD was lower in AC than in non-AC multiples (aOR 0.41, CI 0.20-0.87), and this finding did not change after controlling for mechanical ventilation (aOR 0.42, CI 0.20-0.85) or presence of a patent ductus arteriosus (aOR 0.39, CI 0.18-0.84).

**Conclusion:**

When the analysis is restricted to very preterm infants and stratified by multiplicity, no significant associations between AC and increased risk of short-term mortality and survival without major morbidities emerge. This result is consistent with previous studies, and may confirm the hypothesis that the adverse effects of AC are mediated by preterm birth. However, larger appropriately powered studies are needed before definitely excluding the possibility of adverse events linked to AC in infants born before 32 weeks gestation.

**Electronic supplementary material:**

The online version of this article (doi:10.1186/1471-2393-14-307) contains supplementary material, which is available to authorized users.

## Background

Births after Assisted Conception (AC) have increased in the last decades in industrialized countries, currently accounting for over 1% of all livebirths in the USA, nearly 2% in Italy and UK, and up to 6% in other European countries [1–3]. As a consequence, concerns have been raised about possible increased risks of adverse pregnancy and infant outcomes, in particular after the use of assisted reproductive technologies (ART) that involve gamete manipulation outside the reproductive systems. Multiple pregnancy, placenta praevia, antepartum haemorrhage, incompetent cervix, pregnancy-induced hypertension, preterm birth, low birth weight, foetal growth restriction, birth defects, caesarean delivery, and admission of newborn babies to neonatal intensive care unit (NICU) have been reported [4–7]. Risks appear to be higher in single compared to multiple births [8, 9]. Open issues still remain, mainly related to the potential confounding effects of maternal sociodemographic characteristics and lifestyles, the possibility of spontaneous reduction in case of multifoetal pregnancy, the effect of infertility “per se” and of the different technical approaches adopted [4].

Most of the adverse neonatal outcomes after AC are linked to the increased risk of prematurity. When the analysis was restricted to infants born before 32 weeks gestation (Very Preterm Infants, VPI) or with birth weight <1500 g (Very Low Birth Weight Infants, VLBWI), no excess mortality and morbidity linked to AC was found [10–15]; nor there was an increased emotional burden in mothers after AC during the neonatal period [16]. However, with the exception of one area-based [12] and one multi-centre hospital based [10] studies, most of the evidence is derived from retrospective single-centre investigations [11, 13–16].

In this study we used the data of a large area-based prospective cohort study aiming at comparing the neonatal outcomes of AC- and naturally conceived VPI, separately for singletons and multiples.

## Methods

### Study population

The ACTION (Accesso alle Cure e Terapie Intensive Ostetriche e Neonatali ) project was a prospective area-based cohort study carried out in 6 Italian regions: two in the North (Lombardia and Friuli Venezia-Giulia), three in the Centre (Toscana, Marche and Lazio) and one in the South of Italy (Calabria). Overall, about 40% of all Italian births take place in these regions.

All mothers of infants born at 22 to 31 completed weeks of gestational age (GA) and admitted to NICU between July 2003 and December 2004 in Lombardia, Lazio and Calabria, and up to June 2005 in Friuli Venezia-Giulia, Tuscany and Marche were enrolled in the study (n = 2660). Infants were followed up to discharge from hospital or death in NICU.

The study was approved by the Paediatric Ethics Committee of Tuscany, that was the project-leader region. Parental written informed consent was obtained.

### Data collection

A standardized form was used to collect information on mothers’ characteristics, pregnancy complications and care, babies’ conditions and assistance at birth, morbidities and treatments in the NICU, outcome at discharge from hospital. Assisted conception was defined as any kind of treatment for infertility, including techniques that assist the egg fertilization and implantation. GA was recorded, in completed weeks and days, as the best obstetrical estimate using information on the last menstrual period and ultrasound measures. According to the Italian guidelines, the ultrasound GA estimate was used instead of that based on the date of the last menstrual period when the difference between the two was ≥1 week and the ultrasound evaluation was performed within 16 weeks gestation [17]. The level of centre of birth was defined as tertiary when a 3^rd^ level NICU was present in the same hospital. Complications in pregnancy were defined according to the MOSAIC study [18], and grouped into a) disorders of placentation, including pregnancy hypertension disorders, HELLP syndrome, pre-eclampsia, eclampsia, and foetal growth restriction, and b) intrauterine inflammation/infection, including threatened preterm birth, preterm premature rupture of membranes, antepartum haemorrhage and maternal infection [19]. Small for gestational age (SGA) infants were those whose birth weight was below the 10^th^ percentile, derived from this same cohort by week of gestation and sex. We used the 10^th^ percentile cutoff because it is the most frequently quoted in the literature, and was adopted in other area-based European research projects [18, 20, 21].

Main infants’ outcomes were defined as death before discharge and survival without major morbidities. The last included: intraventricular haemorrhage (IVH, grade 3-4) [22], cystic periventricular leukomalacia (c-PVL, grade 2-4) [23], retinopathy of prematurity (ROP, stage ≥3), and bronchopulmonary dysplasia (BPD, defined as need for oxygen supplementation at 36 weeks GA or at discharge for infants discharged earlier). Other outcomes were ultrasound diagnosed patent ductus arteriosus (PDA), necrotizing enterocolitis (NEC stage 2 or 3, modified Bell’s criteria) [24], any sepsis or meningitis (clinical and hematological signs ± positive culture), length of hospitalization for survivors, and any breastfeeding at discharge.

### Statistical analysis

Single and multiple infants were considered separately.

Univariate chi-squared or t test calculation were used as appropriate to explore the relationships between AC and maternal characteristics, pregnancy complications and infants’ variables.

Multivariable logistic regression models were used to assess the association between AC and infant outcomes, adjusting for potential confounders. Robust variance and standard error estimates of regression parameters were calculated using the clustered sandwich estimator method, to account for the intra-group correlation of observations within NICUs [25]. The following variables were controlled for: gender, any antenatal steroids, GA in completed weeks, SGA status, mode of delivery, region of birth and maternal variables. These included maternal age (coded as <35 or ≥35 years), country of origin (Italy or otherwise), education (primary/lower secondary versus upper secondary/university), and any previous births (yes or no). Missing data in the explanatory variables were included in the models as dummies. Missing values were always ≤ 5%, with the exception of maternal age and education (missing 7% and 14% respectively).

Results are presented in tables as absolute and relative frequencies, and as adjusted odds ratios (aOR) and 95% confidence intervals (CI). Frequencies of neonatal morbidities were computed on infants surviving to diagnostic ascertainment, or to 36 weeks GA age for BPD.

The STATA software, version 10 (StataCorp 2007. Stata statistical software: Release 10. College Station, TX: StataCorp) was used for statistical analyses. Power analysis for multiple logistic regression was performed with the PASS 13 software (NCSS, LLC, Kaysville, Utah, USA) [26].

Data were reported and discussed according to the STROBE statement checklist for observational cohort studies (http://www.strobe-statement.org) (Additional file 1).

## Results

**Figure 1:**
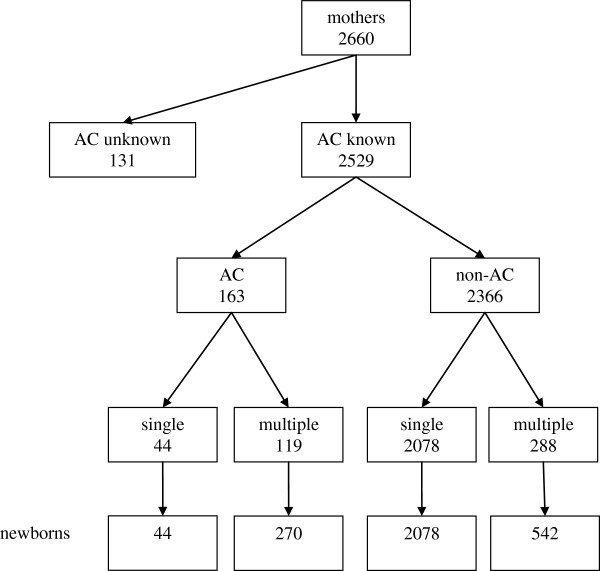
**Description of the cohort under study.**

One hundred and thirty one mothers (4.9%) were excluded because mode of conception was unknown (Figure 1). AC was employed in 163 of the remaining 2529 mothers (6.4%). Details about the technique used were available for 113 births: in vitro fertilization (IVF) or intracytoplasmic sperm injection (ICSI) was used in 68% of them. The overall frequency of multiple births was 16.1% (n = 407): 73.0% (n = 119) in AC group and 12.2% (n = 288) in non-AC group. Liveborn infants were 2934 (314 following AC, 10.7%); 27.7% of them were multiples (n = 812), and 72.3% were singletons (n = 2122).

AC use was significantly more frequent when mothers were older and more educated, and less frequent in foreign mothers and after previous births (Table 1). No statistically significant differences in pregnancy complications, GA distribution and administration of antenatal steroids were found between non-AC and AC pregnancies. Twin, triplet and quadruplet births were more frequent following AC. Mothers with AC pregnancies were more likely to have a spontaneous onset of labour, be delivered in a tertiary centre, and have a caesarean section.

**Table 1:** **Maternal, pregnancy and delivery variables, by mode of conception (n = 2529)**

	Non-AC		AC		***p-value***
	n	(%)	n	(%)	
Age ≥35 years	703	(31.9)	69	(45.4)	*<0.001*
Country of origin other than Italy	541	(23.2)	16	(9.9)	*<0.001*
Upper secondary/university education	1206	(59.6)	108	(78.3)	*<0.001*
Previous births	937	(40.4)	30	(18.5)	*<0.001*
Plurality					
Twin	264	(11.6)	75	(46.0)	
Triplet	14	(0.6)	42	(25.8)	*<0.001*
Quadruplet	0		2	(1.2)	
Complications of pregnancy					
Disorders of placentation^1^	731	(31.4)	46	(28.4)	*0.431*
Inflammation/infection^2^	1446	(61.9)	109	(67.3)	*0.170*
Gestational age at delivery (weeks)					
<26	345	(14.6)	28	(17.2)	*0.606*
26-27	412	(17.4)	23	(14.1)	
28-29	591	(25.0)	39	(23.9)	
30-31	1018	(43.0)	73	(44.8)	
Delivery at tertiary centre	2108	(89.1)	154	(94.5)	*0.031*
Any antenatal steroids	1741	(76.4)	131	(81.9)	*0.111*
Spontaneous onset of labour	1014	(43.8)	84	(52.8)	*0.027*
Caesarean delivery	1687	(71.6)	130	(79.7)	*0.025*

^1^Disorders of placentation include: pregnancy hypertension disorders, HELLP syndrome, pre-eclampsia, eclampsia, foetal growth restriction.

^2^Inflammation/infection disorders include: threatened preterm labor, PPROM, antepartum haemorrhage, maternal infection.

Infants’ characteristics and outcomes by plurality and mode of conception are shown in Table 2. Females were more frequent among AC singletons. Frequencies of mechanical ventilation, PDA, and BPD were lower in AC compared to non-AC multiples. A shorter length of hospitalization was observed in surviving AC multiples.

**Table 2:** **Infants’ characteristics and short term outcomes by plurality and mode of conception (n = 2934)**

	Singletons					Multiples				
	Non-AC (n = 2078)		AC (n = 44)		***p value***	Non-AC (n = 542)		AC (n = 270)		p***value***
	n	(%)	n	(%)		n	(%)	n	(%)	
Gender, females	944	(45.5)	27	(61.4)	*0.036*	255	(47.0)	128	(47.4)	*0.923*
SGA	217	(10.5)	6	(13.6)	*0.498*	41	(7.6)	16	(6.0)	*0.393*
Malformations										
Lethal	2	(0.1)	0			0		0		*0.265*
Acutely life-threatening	35	(1.8)	0		*0.790*	4	(0.8)	0		
Non acutely life-threatening	105	(5.3)	3	(7.1)		23	(4.4)	8	(3.1)	
Apgar score <7/ intubated at 5 m’	965	(47.1)	23	(52.3)	*0.500*	218	(40.7)	101	(38.1)	*0.474*
CRIB score										
>2	773	(41.4)	17	(41.5)	*0.758*	158	(32.8)	77	(32.8)	*0.777*
not attributed	213	(13.2)	3	(6.8)		61	(11.2)	35	(13.0)	
Any surfactant	1164	(57.3)	29	(69.0)	*0.129*	300	(56.7)	139	(53.0)	*0.330*
nCPAP	1462	(72.3)	33	(78.6)	*0.368*	357	(68.3)	167	(64.2)	*0.259*
Mechanical ventilation	1272	(62.7)	31	(72.1)	*0.205*	316	(60.3)	138	(52.5)	*0.036*
PDA	798	(42.5)	20	(55.6)	*0.116*	243	(50.7)	101	(42.8)	*0.046*
NEC	69	(3.5)	0		*0.226*	20	(3.8)	6	(2.4)	*0.286*
Sepsis/meningitis	442	(21.8)	7	(16.7)	*0.425*	106	(20.0)	48	(18.4)	*0.591*
IVH grade 3 or 4	166	(8.5)	6	(14.3)	*0.188*	52	(10.3)	25	(9.9)	*0.865*
c-PVL	99	(5.1)	0		*0.142*	32	(6.23)	12	(4.8)	*0.393*
ROP stage ≥3	79	(4.3)	1	(2.9)	*0.676*	17	(3.7)	5	(2.1)	*0.280*
BPD	219	(12.7)	6	(17.1)	*0.433*	44	(10.1)	9	(4.2)	*0.010*
Death before discharge	364	(17.6)	9	(20.9)	*0.566*	110	(20.4)	52	(19.3)	*0.709*
Survival without major morbidities^1^	1356	(65.4)	26	(60.5)	*0.500*	363	(67.2)	191	(70.7)	*0.310*
Any breast feeding at discharge	1001	(59.8)	23	(74.2)	*0.105*	255	(61.3)	135	(63.4)	*0.611*
Lenght of stay, survivors Days, mean (sd)	64.6	(34.3)	61.9	(31.2)	*0.653* ^*2*^	60.2	(33.2)	54.5	(27.7)	*0.031* ^*2*^

*SGA*: Small for gestational age; *nCPAP*: Nasal continuous positive airway pressure; *PDA*: Patent ductus arteriosus; *NEC*: Necrotizing enterocolitis; *IVH*: Intraventricular haemorrhage; *c-PVL*: Cystic periventricular leukomalacia; *ROP*: Retinopathy of prematurity; *BPD*: Bronchopulmonary dysplasia. Frequencies of morbidities were computed on babies who survived to have the relevant diagnostic ascertainment or up to 36 weeks GA for BPD.

^1^Major morbidities defined as: IVH grade 3-4, c-PVL, ROP stage ≥3, BPD.

^2^t test.

In multivariable analyses (Table 3), the aOR point estimates for hospital mortality and survival without major morbidities were 1.60 and 0.75 respectively in singletons, and 0.79 and 1.30 in multiples. The associations were not significant as all the CIs included unity. However, the statistical power estimates were low, ranging from 0.23 for mortality and 0.13 for survival without major morbidities in singletons, and 0.13 and 0.25 for the two outcomes, respectively, in multiples. We cannot therefore exclude that a much larger study might have led to statistically significant results.

**Table 3:** **Adjusted odds ratios (aOR) of neonatal morbidity and mortality outcomes comparing AC and non-AC babies, separately for singletons and multiples**

	Singletons		Multiples	
	aOR ^1^	95% CI	aOR ^1^	95% CI
PDA	1.63	0.84-3.16	0.64	0.45-0.90
IVH, grade 3-4	2.40	0.93-6.19	1.08	0.52-2.23
c-PVL	- ^2^	-	1.09	0.46-2.58
IVH grade 3-4 or c-PVL	1.36	0.59-3.14	1.00	0.55-1.81
Sepsis/meningitis	0.48	0.20-1.17	0.89	0.53-1.47
NEC	- ^2^	-	0.44	0.19-1.02
ROP, stage ≥3	0.57	0.06-5.51	0.36	0.11-1.15
BPD	1.37	0.58-3.23	0.41	0.20-0.87
Death before discharge	1.60	0.72-3.54	0.79	0.47-1.33
Survival without major morbidities	0.75	0.43-1.30	1.30	0.85-1.99

*PDA*: Patent ductus arteriosus; *IVH*: Intraventricular haemorrhage; *c-PVL*: Cystic periventricular leukomalacia; *NEC*: Necrotizing enterocolitis; *ROP*: Retinopathy of prematurity; *BPD*: Bronchopulmonary dysplasia.

^1^aORs indicate the association between AC and outcomes, adjusting for gender, antenatal steroids, gestational age, SGA status (<10^th^ BW percentile), mode of delivery, region of birth, and mothers’ characteristics (age, country of origin, education, and previous births).

^2^No estimation of the aORs was possible because no cases of c-PVL and NEC were reported for AC singletons.

Similar non-significant differences were found for the other morbidity outcomes in singletons, although in some cases the aOR point estimates were well above 2 (severe IVH) or below 0.5 (sepsis/meningitis). Among multiples, however, statistically significant lower risks for PDA (aOR 0.64, CI 0.45-0.90) and BPD (aOR 0.41, CI 0.20-0.87) were found, while for NEC the decreased risk (aOR 0.44, CI 0.19-1.02) was at borderline significance. The association between AC and PDA in multiples remained statistically significant after adjustment for mechanical ventilation (aOR 0.68, CI 0.48-0.97), and that with BPD did not change after controlling for ventilation or PDA, (aORs 0.42, CI 0.20-0.85, and 0.39, CI 0.18-0.84, respectively).

## Discussion

In this area-based cohort of very preterm infants we could not demonstrate clear differences in mortality and survival without major morbidities between naturally and AC-conceived babies.

To some extent our findings are comparable to those of previous studies in very preterm or VLBWI. Stewart et al. [10] studied 1473 VLBWI, and found no difference in cranial ultrasound abnormalities between naturally conceived babies and those born after ART or use of fertility therapy. In multiple VLBWI Hashimoto et al. [11] found no difference in mortality, BPD, and the combined outcome mortality or BDP between naturally conceived babies and those conceived following fertility treatment. In a large population of VLBWI, Schimmel et al. [12] found no difference in mortality, respiratory distress syndrome, PDA, NEC, IVH, BPD, and congenital malformations between babies born after IVF (n = 1396) and those naturally conceived (n = 6765). Messerschmidt et al. [13] compared 195 VLBWI born after IVF with 1228 naturally conceived VLBWI, and found no difference in mortality, short term pulmonary morbidity, severe cerebral morbidity and frequency of SGA babies. Shah et al. [14] compared the neonatal outcome of 137 multiple infants ≤32 weeks GA born after ART with that of 233 naturally conceived multiple babies with similar GA, and found no difference in outcome defined as a combination of death, grade 3/4 IVH or periventricular leukomalacia, ROP stage >2, and chronic lung disease. Picaud et al. [15] reported survival without severe morbidity in 612 infants <33 weeks GA: 81 were born following ART and 521 were naturally conceived. Using a composite morbidity index based on the occurrence of NEC, IVH grade ≥3, periventricular leukomalacia, and BPD, these authors found that survival without severe morbidity was higher in ART infants.

Most of the unfavourable neonatal outcomes reported when also moderate/late preterm and term infants are included in the study populations are probably the consequence of the shift of GA distribution toward lower values; this shift is predominantly related to the higher frequency of multiplicity, but it was observed also in single pregnancies [4, 8, 27–29]. Causes of infertility, in adjunct to and independently from factors related to the reproductive technology itself, may also contribute to the unfavorable outcomes associated with AC [30]. However, when only very preterm or very low birth weight infants are considered, the effect of multiplicity on gestational age does not apply, and both AC- and naturally conceived infants are exposed to the adverse influence of very early birth and immaturity of organs. Additionally, AC pregnancies may benefit from the better prenatal and perinatal care associated with medically assisted reproduction, including earlier contact with the obstetric services and more careful monitoring of mother and foetus conditions [15, 31]. It is also possible that pre-pregnancy health and lifestyles of mothers determined to conceive and ultimately accessing AC services are more favourable than in naturally conceiving women and able to promote better perinatal outcomes [32].

Indeed in our study women using AC, although older than those who conceived naturally, did not seem to have increased rates of pregnancy inflammation/infection or disorders of placentation that have been linked with different patterns of placental pathologies and foetal/neonatal outcomes [19, 33, 34]. They were also more likely to be Italian and to have a higher education, and therefore have access to the information and contacts that facilitate healthier lifestyles and better health care.

We found that, compared to non-AC peers, AC multiples had a lower risk of BPD, and this result persisted after adjustment for PDA and mechanical ventilation, two possible antecedents of BPD. Previous studies reported inconclusive results about BPD. Schimmel et al. found no difference in risk of chronic lung disease in single and multiple VLBWI according to mode of conception [12], while Picaud et al. reported a lower BPD rates in infants <29 weeks gestation born after ART [15]. In contrast, Shah et al. found higher rates of BPD in ART preterm multiples less than 33 weeks gestation compared to naturally conceived peers [14].

These results are not directly comparable with ours because of differences in study design (centre-based rather than area-based) or eligibility criteria (birth weight rather than gestational age). Other studies including both preterm and at-term twins found better perinatal outcomes in ART- than in naturally conceived infants [31, 35]. Most unlikely these differences can be causally linked to specific ART treatment effect. As already discussed, better pre-pregnancy health and perinatal care associated with medically assisted reproduction may result in offspring with more favourable clinical conditions.

This study has limitations. As in most studies focusing on the special populations of very preterm or very low birth weight infants (about 1-1.5% of total births), sample size may be not large enough to detect the neonatal outcomes associated with AC. Also, we were not able to separately investigate the risks of the different AC technologies, particularly those involving manipulation of gametes outside the reproductive system, such as IVF and ICSI. Nevertheless, the worse outcome in AC than in non-AC singletons suggested by our findings, and the reverse trend in multiple babies, is in agreement with the worse outcomes in AC-conceived singletons reported in recent meta-analyses [29, 36].

The strengths of this study are the area-based prospective design, thus averting selection biases caused by a centre-based approach. Additionally, and unlike most previous studies, recruitment was based on GA, which prevented the bias arising when the birth weight criterion is employed to study the relationship between antenatal and perinatal factors and neonatal outcomes.

## Conclusion

In our population of newborn infants <32 weeks GA, although a lower risk of some short-term outcomes, such as PDA and BPD, was found in AC-multiples, we could not demonstrate the presence or absence of different risks of mortality and survival without major morbidities in AC compared to non-AC conceived babies. A final conclusion about differences of outcomes in VPI born from assisted conception or naturally conceived cannot therefore be reached. Although some of our results may be of interest and suggest clues for clinical interpretations, larger and more powered studies are needed before confidently exclude the effects of AC on the short term outcomes of very preterm infants. To this end, this study can serve as a suitable pilot investigation.

## Electronic supplementary material

Additional file 1: **STROBE Statement—Checklist of items that should be included in reports of cohort studies.** (DOC 90 KB)

## Abbreviations

AC: Assisted conception
ART: Assisted reproductive technology
NICU: Neonatal intensive care unit
VPI: Very preterm infants
VLBWI: Very low birth weight infants
GA: Gestational age
PDA: Patent ductus arteriosus
NEC: Necrotizing enterocolitis
IVH: Intraventricular haemorrhage
c-PVL: Cystic periventricular leukomalacia
ROP: Retinopathy of prematurity
BPD: Bronchopulmonary dysplasia
SGA: Small for gestational age
IVF: In vitro fertilization
ICSI: Intracytoplasmic sperm injection.
